# Solitary Orbital Metastasis 35 Years after a Diagnosis of Lobular Carcinoma in Situ

**DOI:** 10.7759/cureus.1404

**Published:** 2017-06-28

**Authors:** Matthew B Spraker, Courtney E Francis, Larissa Korde, Janice Kim, Lia Halasz

**Affiliations:** 1 Department of Radiation Oncology, University of Washington, Seattle, WA; 2 Department of Ophthalmology, University of Washington, Seattle, WA; 3 Divison of Oncology, University of Washington, Seattle, WA; 4 Department of Radiation Oncology, University of Washington, Seattle, Wa

**Keywords:** breast, metastasis, orbit, protons, radiotherapy, eye, aromatase inhibitor

## Abstract

This report describes a solitary orbital metastasis of lobular carcinoma in a woman 35 years after diagnosis of right breast lobular carcinoma in situ (LCIS). After partial response to anastrozole, the patient was treated with proton radiotherapy to 45 cobalt gray equivalents (CGE) with an excellent response. We additionally discuss treatment strategies for this rare metastatic site.

## Introduction

Orbital metastases (OM) are a rare manifestation of systemic disease. Patients commonly present with diplopia and pain [1-2]. Breast cancer is the most common histology associated with OMs [1, 3], with invasive lobular carcinoma being more common than ductal disease [3]. Here we present a case of a new solitary orbital metastasis in a woman who was disease-free for 35 years after a prior diagnosis of right breast lobular carcinoma in situ (LCIS).

## Case presentation

A 75-year-old woman with a past medical history of right breast lobular carcinoma in situ (LCIS) presented with a three-month history of right eye swelling and diplopia. On exam, she was noted to have periorbital edema and erythema, ptosis, and enophthalmos. She had limited ocular movements and small angle right hypotropia in primary gaze. There was no evidence of optic neuropathy. Neurologic exam was normal. Clinically, there was no evidence of recurrence in the breast, lymphadenopathy, or bone metastases. Biopsy revealed individual neoplastic cells infiltrating fibroadipose tissue in a linear pattern. Immunochemistry was positive for estrogen receptor (ER) expression and negative for progesterone (PR) receptor. Fluorescence in situ hybridization was negative for human epidural growth factor receptor 2 (HER2) gene amplification. Pathology was reported as invasive carcinoma, consistent with a mammary primary and suggestive of lobular carcinoma.  

Past medical history included a reported diagnosis of LCIS diagnosed 35 years ago after she presented with a right breast lump. She was treated with a bilateral nipple-sparing mastectomy with immediate implant reconstruction. Unfortunately, tissue from the initial LCIS tumor was not available for review. Since that time, she has had her implants replaced twice without any complications or suspicion of recurrence. She has had no symptoms or signs of breast cancer recurrence and has had negative surveillance mammograms.

**Figure 1:**
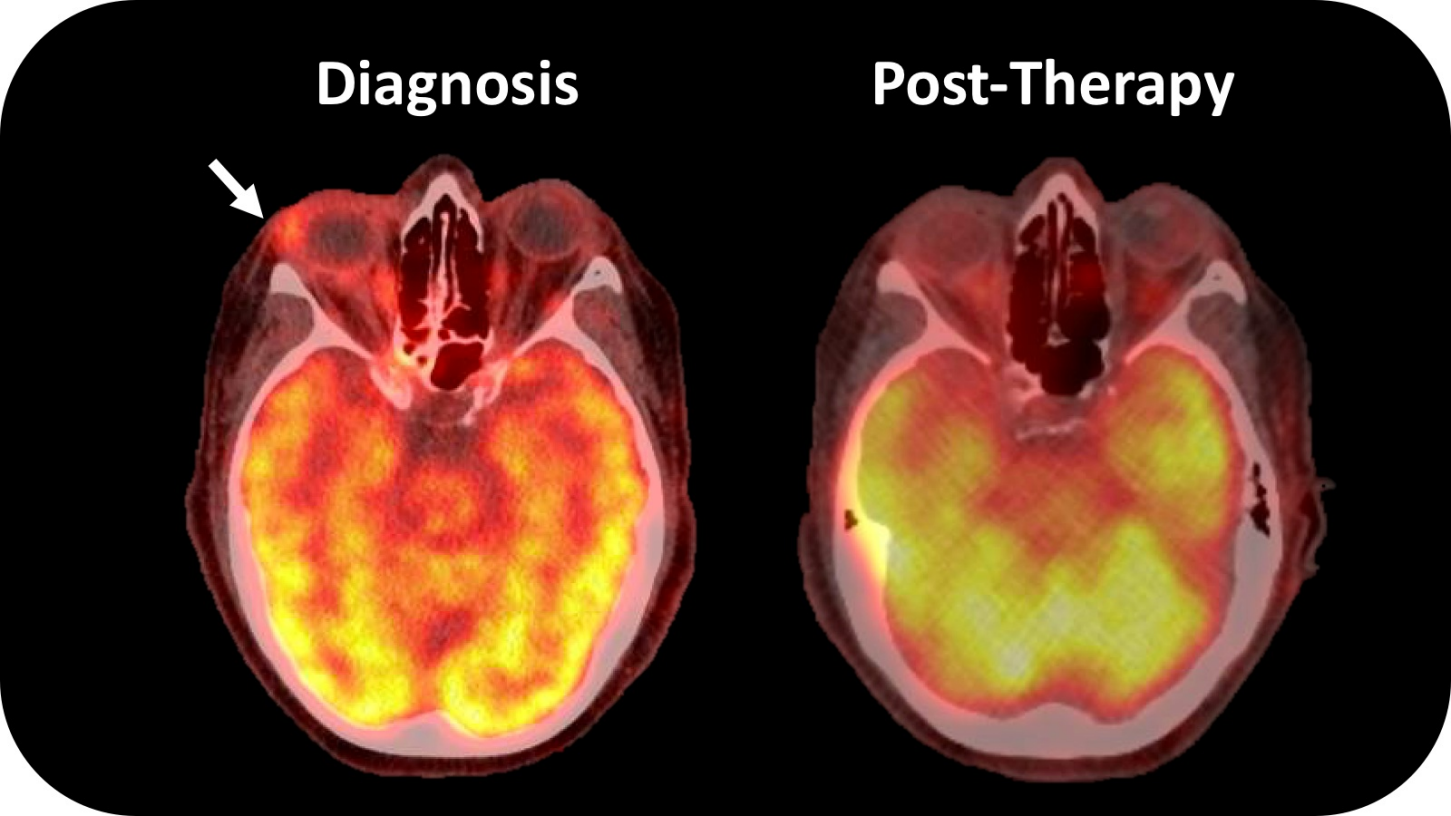
Positron emission tomography–computed tomography of the orbits at diagnosis and post-treatment Depicts PET/CT of the orbits at diagnosis and 11 months after anastrozole and proton radiotherapy. The PET/CT at diagnosis demonstrates diffuse abnormal uptake with focal areas of high standardized uptake (white arrow). After treatment, the generalized uptake is decreased and the focal areas are resolved.

Magnetic resonance imaging (MRI) of the orbits demonstrated enhancement and edema of the extraocular muscles of the right eye and mild optic neuritis. Positron emission tomography and computed tomography (PET/CT) demonstrated generalized increased radiotracer uptake in the soft tissue of the right globe (Figure 1). There was no other evidence of metastatic disease.

**Figure 2:**
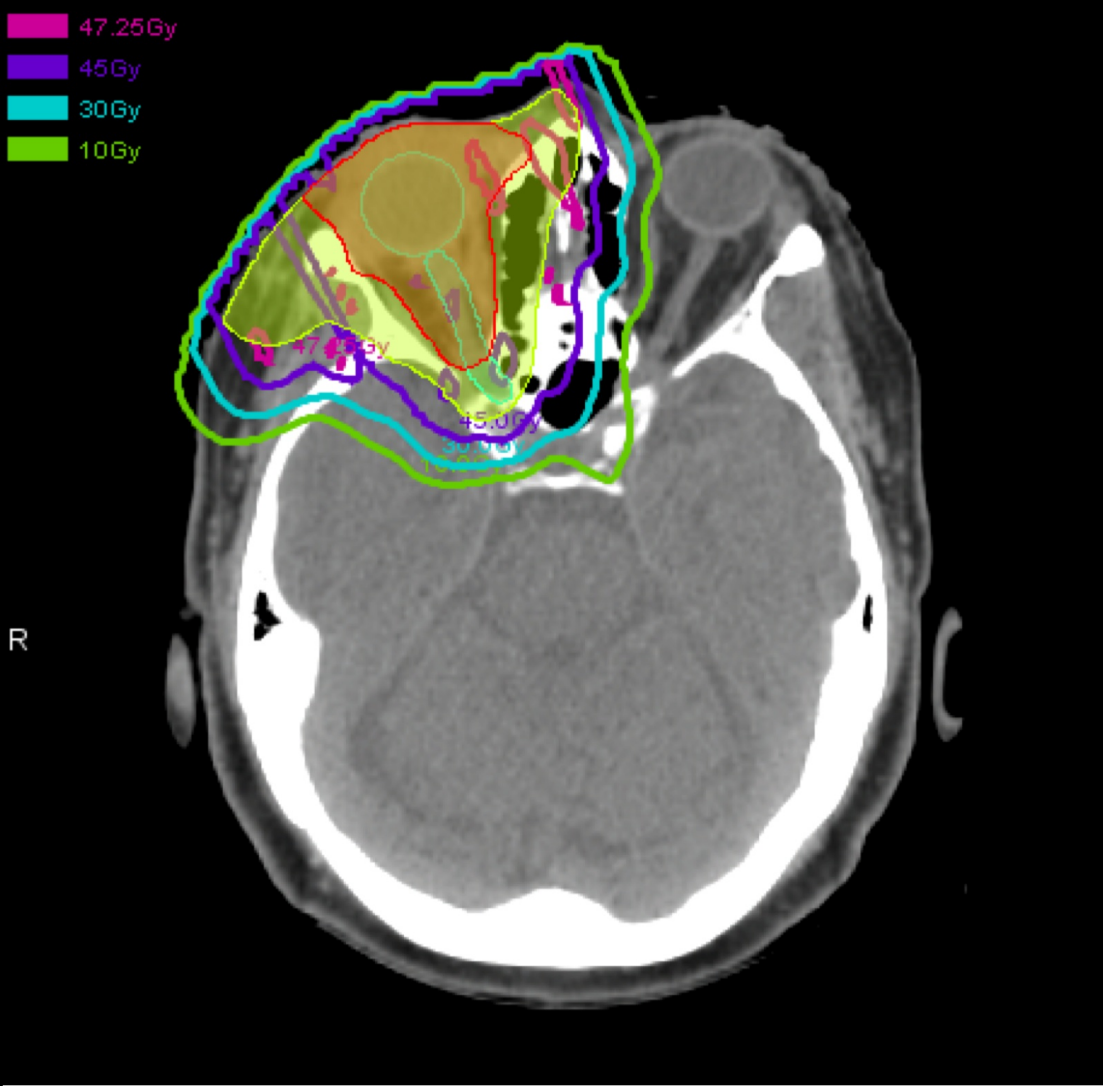
Proton therapy dosimetry plan The clinical target volume (CTV, red) and planning target volume (PTV, yellow) are surrounded by the precription dose line, 45 cobalt gray equivalents. Dose to the contralateral eye was minimized.

The patient was treated with anastrozole (one mg daily) for eight weeks with minimal improvement. She was then treated with proton radiotherapy to 45 cobalt gray equivalents (CGE) in 25 fractions delivered over 30 days. The radiation field is shown in Figure 2. The clinical and planning target volumes encompass the diffuse area of enhancement on MRI and hyperactivity on PET/CT. The dose was constrained to 45 CGE maximum for the right globe. The radiotherapy plan was designed to keep the dose to the left eye, optic nerves, and brain as low as possible, given her right vision changes and low volume, oligometastatic disease. The side effects of the treatment included tenderness, acute erythema, and skin desquamation at the end of treatment, which required antimicrobial therapy due to concern for cellulitis. There was no change in visual acuity.

An MRI four months after radiation treatment showed stable thickening but decreased contrast enhancement of the periorbital soft tissues, and a PET scan at 11 months after radiation treatment showed reduced radiotracer uptake in the right orbit compared to the exams prior to treatment (Figure 1). She was last seen in follow-up 16 months following the completion of radiotherapy. Extraocular motility has improved, but she remains with a stable, small, angle right hypotropia that is well corrected with prism glasses. Ptosis and enophthalamos are stable. There are no signs of optic neuropathy or retinopathy. She continues anastrozole with no additional evidence of metastatic disease on surveillance exams and PET/CT.

## Discussion

Breast cancer is the most common presenting histology of OMs, comprising 16% to 57% of patients in published case series [1-3]. OMs are five times more likely to be lobular carcinoma than ductal carcinoma or other breast histologies [3]. Interestingly, the orbital fat pad is a common presenting sub-site for OMs [1], and it has been suggested that fat pad hormone production may explain the predilection of invasive lobular carcinoma to metastasize to the orbit [3]. 

LCIS is not regarded as a malignant disease, so it is noteworthy that the patient presented with a solitary orbital metastasis after a 35-year disease free interval. The original breast tumor tissue was not available for evaluation, so it is possible that an invasive component or second tumor was not appreciated. Alternatively, the patient could have developed a sub-clinical second primary in residual breast tissue after a nipple-sparing mastectomy.

The literature supports the use of hormonal therapy for OMs. Prior series have shown regression of choroidal [4] and uveal [5] breast cancer metastases after treatment with aromatase inhibitors. While this patient had partial radiographic improvement with anastrozole, she had only minimal clinical improvement. Given persistent symptoms and oligometastatic disease, we recommended radiotherapy. One report describes radiotherapy treatment of 28 patients with eye metastases to a median dose of 3980 cGy in 20 fractions with a cobalt-60 (Co-60) unit [6]. A palliative dose of 3500-5000 cGy in conventional fractionation for OMs is recommended, depending on the goals of treatment. Doses to the lacrimal gland and anterior structures of the eye should be limited where possible.

While hypofractionated regimens are discouraged due to the risk of late toxicity [1], advancements in particle and image-guided therapy now allow for safe, effective treatment of orbital tumors. Proton therapy was used in this case to maximize dose homogeneity and achieve maximal sparing of contralateral eye, optic nerves, and normal brain.

## Conclusions

This case outlines an unusual presentation of isolated metastasis and emphasizes the multidisciplinary approach to treatment that our group pursued for this patient. Ultimately, she had an impressive response to combined treatment with anastrozole and proton therapy with minimal side effects. Long-term surveillance will be required to determine whether she continues to remain in remission and for long term side effects. 
